# RF Thermal Plasma Synthesis of Ultrafine ZrB_2_-ZrC Composite Powders

**DOI:** 10.3390/nano10122497

**Published:** 2020-12-12

**Authors:** Liuyang Bai, Fangli Yuan, Zheng Fang, Qi Wang, Yuge Ouyang, Huacheng Jin, Jiaping He, Wenfu Liu, Yinling Wang

**Affiliations:** 1 School of Mechanical and Energy Engineering, Huanghuai University, Zhumadian 463000, China; fangyyyy2020@163.com (Z.F.); wq19853448069@163.com (Q.W.); liuwenfu@huanghuai.edu.cn (W.L.); wangyinling@huanghuai.edu.cn (Y.W.); 2 Institute of Process Engineering, Chinese Academy of Sciences, Beijing 100190, China; flyuan@home.ipe.ac.cn (F.Y.); ouyangyuge@btbu.edu.cn (Y.O.); hcjin@ipe.ac.cn (H.J.); hejiaping@msn.com (J.H.); 3 College of Chemistry and Materials Engineering, Beijing Technology and Business University, Beijing 100048, China

**Keywords:** ZrB_2_, ZrC, composite powders, RF thermal plasma, chemical synthesis

## Abstract

Ultrafine ZrB_2_-ZrC composite powders were synthesized via a radiofrequency (RF) thermal plasma process. Numerical simulation and thermodynamic analysis were conducted to predict the synthesis process, and experimental work was performed accordingly to demonstrate its feasibility. The as-prepared samples were characterized by XRD, FESEM, particle size analyzer, nitrogen/oxygen analyzer, Hall flowmeter, and the Brunner-Emmet-Teller (BET) measurements. The thermodynamic analysis indicated that ZrB_2_ was preferentially generated, rather than ZrC, and numerical simulation revealed that the solid raw materials could disperse well in the gaseous reactants, and experimental work showed that free carbon particles were easily removed from the products and the elements of Zr, B, C, and O exhibited a uniform distribution. Finally, ZrB_2_-ZrC composite powders with a particle size of about 100 nm were obtained, the surface area of which was 32.15 m^2^/g and the apparent density was 0.57 g/cm^3^.

## 1. Introduction

Borides and carbides of zirconium show a number of excellent properties, such as high melting point, low density, good wear resistance, high thermal and electrical conductivity, and good chemical stability, which makes them attractive candidates in the areas where wear–corrosion–oxidation resistance is demanded, such as high temperature electrodes, molten metal crucibles, thermal protection systems for hypersonic flights, atmospheric re-entry vehicles, rocket propulsion systems, and nose caps [1,2,3,4,5,6,7,8]. However, it is difficult to use single phase material to meet all the necessary requirements demanded by the extreme conditions encountered in aerospace engineering. Therefore, research efforts have now turned to the development of double or multiple phase ceramics [9,10,11,12]. It has been demonstrated that ZrB_2_-ZrC composites possess better properties, such as mechanical strength and oxidation resistance, than those of either individual component [13,14,15,16,17,18]. Sorrell reported that boride–carbide eutectic composites fabricated by a directional solidification method had very good physical and chemical properties [19]. Tsuchida and Yamamoto synthesized a ZrB_2_-ZrC composite by mechanically activating self-propagating high temperature synthesis (SHS) in a Zr/B/C elemental system [20]. 

During the past decade, there has been a growing appeal for the production of ceramic powders with ultrafine or nano-sized particles due to their excellent sintering ability and the improved mechanical properties of the formed nanograined materials [21,22,23]. The plasma synthesis process is one of the most efficient methods for producing ultrafine powders [24,25,26]. In the plasma system, a high-temperature flame (up to 10,000 K) provides enough energy for the evaporation of the raw materials and the high-temperature gradient helps to solidify the products rapidly, which is necessary for the formation of nanoparticles. The radiofrequency (RF) thermal plasma gas does not come into contact with electrodes and the operation environment is flexible from oxidizing to reducing atmosphere, which can eliminate possible sources of contamination.

RF thermal plasma for the powder synthesis originated from the spheroidization. High melting point metals, ceramic, or irregular-shaped glass micron powders were injected from the top of the plasma torch and heated rapidly in the plasma region, and subsequently cooled down at a high rate when they flew out of the plasma flame, and finally condensed to very fine spherical powders [27,28]. For the synthesis of nanopowders, there are two different ways known as physical vapor deposition (PVD) and chemical vapor deposition (CVD). In a typical plasma synthesis process, the raw materials evaporate in the high-temperature region, then react (if there are chemical reactions involved), and finally condense into fine particles. Hou synthesized single-crystalline α-Si_3_N_4_ nanospheres with a uniform size of ∼50 nm using coarse silicon nitride (5–10 μm) as starting powders in a one-step and continuous way [29]. Kim demonstrated scalable manufacturing of boron nitride nanotubes (BNNTs) directly from hexagonal BN (hBN) powders by using induction thermal plasma, with a high-yield rate approaching 20 g/h [30]. Kumar reported a new ultrafast gas-phase method for synthesizing highly crystalline titanate phase nanowires (NWs) using oxidation of either Ti metal (foils or powders) or spherical TiO_2_ powders with an atmospheric pressure microwave plasma [31]. It can be seen that the reaction system and powder composition were usually simple during early research. 

Recently, there have been reports referencing complicated compounds and reactions. Westover demonstrated the theoretically guided plasma synthesis of high purity nanocrystalline Li_3.5_Si_0.5_P_0.5_O_4_ and fully amorphous Li_2.7_Si_0.7_P_0.3_O_3.17_N_0.22_ [32]. ZrB_2_ and ZrC nanopowders were successfully synthesized via vapor chemical deposition and the metallothermic synthesis route within the RF thermal plasma system, respectively [33,34].

In the present work, ZrB_2_-ZrC composite powders were synthesized via the thermal plasma route, in which ZrCl_4_, B, Mg, and CH_4_ were used as raw materials. Numerical simulation and thermodynamic analysis were conducted ahead to predict the synthesis process, and experimental work was performed accordingly to demonstrate its feasibility. The as-prepared samples were characterized by XRD, FE-SEM, particle size analyzer, nitrogen/oxygen analyzer, Hall flowmeter, and BET method. It is be demonstrated in this work that ultrafine ZrB_2_-ZrC composite powders can be synthesized successfully via the RF thermal plasma technique.

## 2. Materials and Methods 

### 2.1. Materials

ZrCl_4_ (Zr + Hf ≥ 38%) was supplied by Yixian Jincheng Zirconium Industry Company (Jinzhou, China). B (95%) was supplied by Dandong Chemical Engineering Institute Company (Dandong, China). CH_4_ (99.999%), H_2_ (99.999%), and Ar (99.99%) were supplied by Qianxi Jingcheng Gas Industry Company (Beijing, China). Hydrochloric acid (36–38 wt.%) was supplied by Beijing Chemical Reagents Company (Beijing, China).

### 2.2. Synthesis

**Figure 1:**
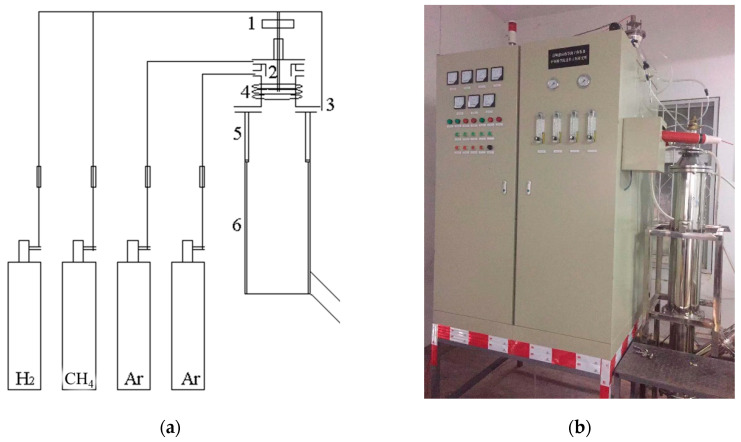
Schematic illustration (**a**) and picture (**b**) of the radiofrequency (RF) thermal plasma processing system: (1) powder feeder; (2) injection probe; (3) circular nozzle; (4) water-cooled induction coil; (5) hot-chamber reactor; (6) cooling-chamber reactor.

Figure 1 presents the schematic illustration and a picture of the RF thermal plasma processing system used in our laboratory, which consists of an RF generator, a plasma generator, a downward plasma torch, a 3-coil cylindrical reactor, a quenching chamber, a precursor feeding system, a powder-collecting filter, a gas supplier system, and an off-gas exhaust system. The reactor is operated in atmosphere and the pressure is usually adjusted in the range of −500 to −1000 Pa gauge during the operation of experiments. The RF generator (30 kW, 4 MHz) was supplied by Tieling High Frequency Equipment Factory, and the reactors were homemade in the Institute of Process Engineering, Chinese Academy of Sciences.

The raw materials of ZrCl_4_, B, and Mg were sifted through a 150 μm sieve and blended in a mechanical mixer. The reactor system was first purged with Ar to remove oxygen and moisture from the reactor, so that a separate inert atmosphere was prepared for the chemical synthesis procedure. A Plasma flame was generated using argon as both the plasma-forming gas and sheath gas. The reactor was heated by the plasma flame for about 5 min until the system reached a steady level, and then the raw materials were injected into the plasma flame in a continuous way with a homemade screw feeder. CH_4_ and H_2_ (if necessary) were mixed into the carrier gas and fed axially through the injection probe to the top of the plasma flame. The typical processing parameters for RF thermal plasma in our laboratory are given in Table 1.

**Figure 2:**
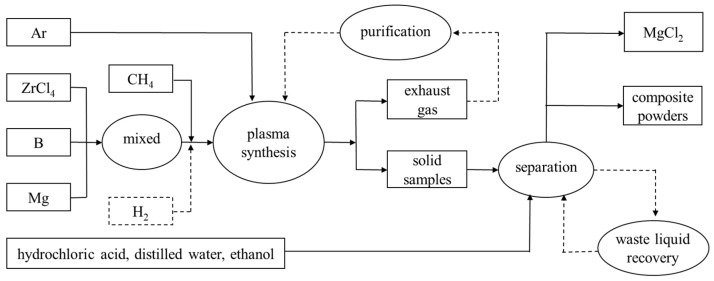
Process flow chart for plasma synthesis of ultrafine ZrB_2_-ZrC composite powders.

For the synthesis of the ZrB_2_ powders, stoichiometric amounts of ZrCl_4_, B, and Mg were weighed with a 20% excess amount of Mg, in which the molar ratio of Zr/B/Mg/C = 1/2/2.4/0–0.1. For the synthesis of the ZrB_2_-ZrC composite powders, the mole ratio of Zr/B/Mg/C = 1/1/2.4/0.5. The feeding time was usually 10–20 min, and then the plasma flame was shut down. In order to protect the products from oxidizing at the high temperature, Ar was supplied continuously until the reactor system cooled down. The final products were collected from the powder-collecting filter, and a small part of them could be found at the bottom of the collector. The solid products needed further post-treatment with diluted hydrochloric acid. The process flow chart is provided in Figure 2.

### 2.3. Characterization

The crystalline phase of the as-prepared intermediate and final products was characterized by an X-ray diffractometer (XRD, X’pertPRO, Panalytical, Almelo, The Netherlands) in a 2θ range from 10° to 90°. Their size and morphology were inspected with field emission scanning electron microscopy (FESEM, JSM-6700 F; JEOL, Tokyo, Japan). The particle size distribution was measured using a LS particle size analyzer (Beckman Coulter LS 13 320, Nyon, Switzerland). The oxygen content was measured by an impulse-thermal conductivity method (Eltra ON-900, NCS, Beijing, China). The carbon content was measured by an infrared C-S measurer (NCS CS-3000, NCS, Beijing, China). The specific surface area was determined using a BET method. The apparent density was examined with the help of a Hall flowmeter.

## 3. Results and Discussion

### 3.1. Thermodynamic Calculation

The plasma synthesis of ZrB_2_ using ZrCl_4_, B, and Mg is ruled by the following reactions:

ZrCl_4_ + 2B + 2Mg → ZrB_2_ + 2MgCl_2_ (1)

ZrCl_4_ + CH_4_ → ZrC + HCl (2)

Main reactions during the plasma processing can be expressed as below:

ZrCl_4_ + 2Mg → Zr + 2MgCl_2_ (3)

CH_4_ → C + 2H_2_ (4)

ZrCl_4_ + 2H_2_ → Zr + 4HCl (5)

Zr + 2B → ZrB_2_ (6)

Zr + C → ZrC(7)

**Figure 3:**
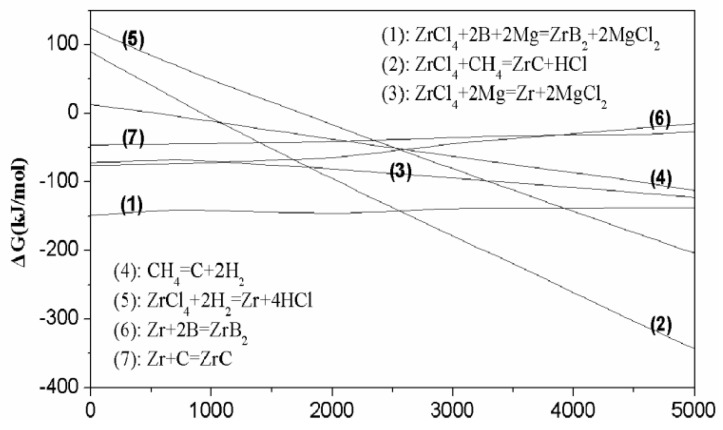
Temperature dependences of the free energy of formation.

The spontaneous direction of a reaction is usually judged by the change of the Gibbs-free energy ΔG. Thermodynamic calculation has been made based on the data given in the literature [35]. Figure 3 shows the free energies of the above reactions as a function of temperature.

ΔG value for reaction (1) is much lower than zero at the temperature, and the ΔG value for reaction (2) decreases as the temperature increases and becomes lower than zero at the temperature around 1000 °C, indicating both reactions could take place spontaneously at high temperature. ΔG value for reaction (6) is always lower than that of reaction (7) at a temperature below 3800 °C, indicating that ZrB_2_ is preferentially generated. Therefore, free C instead of B would exist in the resulting products when C and B in the starting materials excess the stoichiometric ratio.

### 3.2. Numerical Simulation

**Figure 4:**
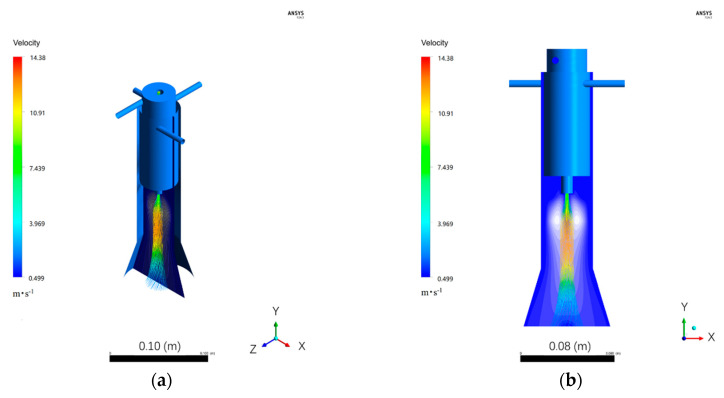

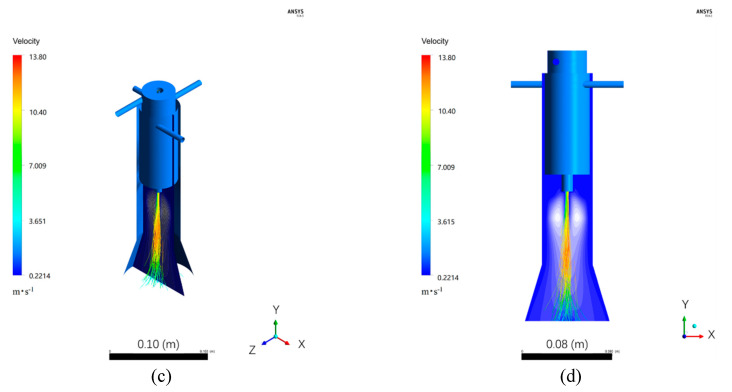
Velocity field simulated by fluent: (**a,b**): carrier gas path lines; (**c,d**): particle tracks.

Fluent software is employed to simulate the system and analyze the flow field and particle trajectories in the reactor as reported in [36]. In the present work, a three-dimensional physical model is applied, and mass-flow-inlet boundary was selected. Figure 4 shows the velocity field. Figure 4a,b shows the carrier gas path lines and Figure 4c,d shows the particle tracks from different points of view. It can be seen that both the carrier gas and particles are well fixed at the central area in the reactor. Their main swelling parts exhibit a similar shape, and the carrier gas expands a bit wider than the particles. These simulation results indicate that the addition of CH_4_ to the (ZrCl_4_ + 2B + 2Mg) reaction system can form another well-distributed system.

### 3.3. Experimental Results

ZrB_2_ and ZrC can be synthesized using (ZrCl_4_ + 2B + 2Mg) and (ZrCl_4_ + CH_4_) reaction systems, respectively, as reported in our previous work [33,34]. Otherwise, ZrC was also synthesized successfully using (ZrCl_4_ + C + 2Mg) reaction system, which is similar as the synthesis of ZrB_2_ reported in [33]. In order to avoid the possible contamination of oxygen residual or leaking in the reaction system for the solid-state synthesis (ZrCl_4_ + 2B + 2Mg or ZrCl_4_ + C + 2Mg), hydrogen was mixed into the carrier gas, so that low oxygen content can be achieved.

In the present work, the two reactions (ZrCl_4_ + 2B + 2Mg) and (ZrCl_4_ + CH_4_) coexist in one system, where solid raw materials including ZrCl_4_, B, and Mg were mixed in advance and CH_4_ was mixed into the carrier gas which carried the solid raw materials into the plasma flame through the axial injection probe.

**Figure 5:**
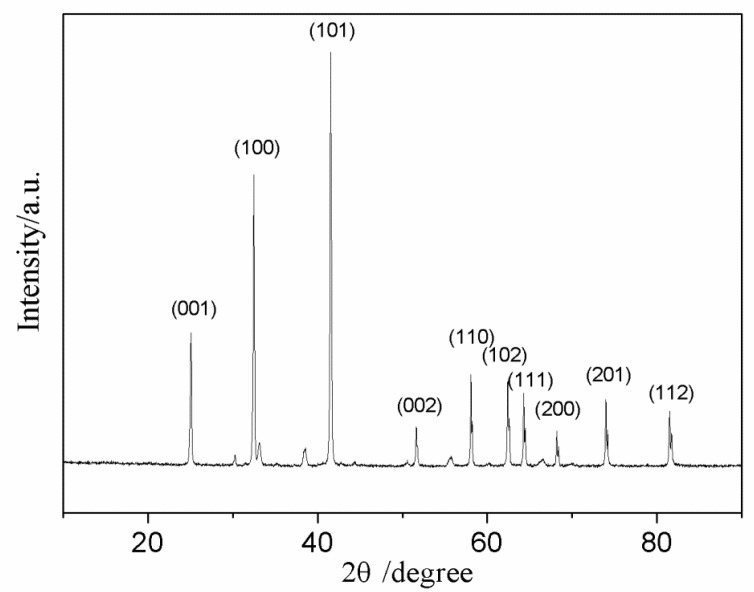
XRD pattern of the sample obtained when the mole ratio of B/Zr was set at 2.0.

The ratio between CH_4_, B, and ZrCl_4_ had great effects on the composition of the products. At the beginning of the experimental work, the mixed raw materials for the synthesis of ZrB_2_ powders were used, in which stoichiometric amounts of ZrCl_4_, B, and Mg were weighed with a 20% excess amount of Mg. Figure 5 shows the XRD pattern of the sample obtained with the mole ratio of CH_4_/ZrCl_4_ of 10%. 

The diffraction peaks of (001), (100), (101), (002), (110), (102), (111), (200), (201), and (112) of a face-centered cubic (fcc) ZrB_2_ (JCPDS 00-034-0423) can be clearly observed in the XRD pattern shown in Figure 5. Furthermore, no obvious peaks of ZrC are displayed in the XRD pattern, which is also inconsistent with the conclusion that ZrB_2_ will be preferentially generated, from thermodynamic analysis.

In the present synthesis process, with a mole ratio of B/Zr of 2, the element B was sufficient and there was little chance of C combining with Zr. Therefore, excessive CH_4_ in the plasma reaction system led to free C in the plasma products. It was observed that there were some black samples floating on the surface of the solution during the post-treatment. The black samples were collected and dried at 110 °C for 120 min. Then, the black samples were confirmed as free C with an infrared C-S measurer, which could be removed from the products easily during the post treatment due to its light weight. Moreover, the C content of the final products measured was quite low. Table 2 displays the carbon contents and oxygen contents of the final products after acid leaching synthesized with a different mole ratio of CH_4_/ZrCl_4_. 

C content was only 0.32% when the mole ratio of CH_4_/ZrCl_4_ was as high as 1/10. In order to demonstrate the separation efficiency of carbon nanoparticles, the sample after acid leaching was filtered and washed without removing the floating black samples on the surface of the solution and characterized using an infrared C-S measurer. The total C content was determined as 1.06%, which was much higher than that of the sample obtained when getting rid of the floating black samples. In consideration of the XRD characterization results, C mainly exists as free carbon instead of combined carbon because no peaks of ZrC are detected in the XRD pattern.

However, the addition of CH_4_ to the (ZrCl_4_ + 2B + 2Mg) reaction system can help to reduce the oxygen content of the final products. The decomposition products of CH_4_, H_2,_ and free C would provide a stronger reduction environment and protect the reactants from oxidation. The sealing technique at the ultra-high-temperature region within plasma equipment is still a challenge. The addition of reducing gas to protect the reaction process can really contribute to the synthesis of low oxygen content products.

**Figure 6:**
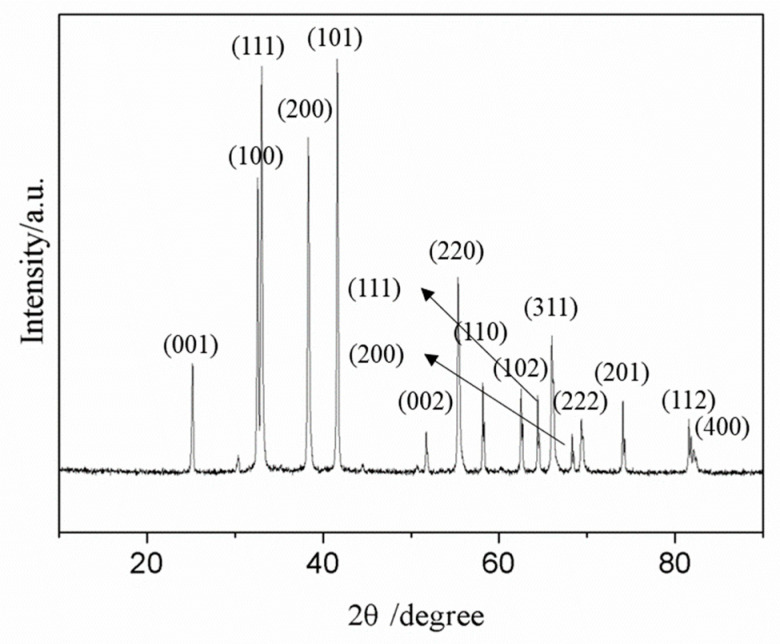
XRD patterns of the samples obtained when the mole ratio of B/Zr was set at 1.0.

In order to get ZrB_2_-ZrC composite powders, the mole ratio of B/Zr was set at 1.0, and the mole ratio of C/Zr was set at 0.5. Figure 6 shows the XRD patterns of the sample, in which both diffraction peaks of (001), (100), (101), (002), (110), (102), (111), (200), (201), and (112) of face-centered cubic (fcc) ZrB_2_ (JCPDS 00-034-0423) and diffraction peaks of (111), (200), (220), (311), (222), and (400) of face-centered cubic (fcc) ZrC (JCPDS 03-065-0332) can be observed, indicating that the obtained products were ZrB_2_-ZrC composite powders.

Carbon and oxygen contents were also measured and are listed in Table 3. Carbon content is 5.29% when the mole ratio of C/B/Zr in the starting materials is set at 1:2:2. The theoretical content of C in ZrC and B in ZrB_2_ is 11.6% and 19.1%. Accordingly, the theoretical content of C in the ZrB_2_-ZrC composite powders (the mole ratio of ZrB_2_/ZrC: 1/1) is 5.6%, which is slightly higher than that detected by the impulse-thermal conductivity method. It can be seen that the oxygen content is 3.5%, indicating that some Zr exist as oxides instead of borides or carbides. Therefore, CH_4_ was not fully utilized to form ZrC. Part of CH_4_ was decomposed into free C and removed from the products during the post-treatment procedure.

**Figure 7:**
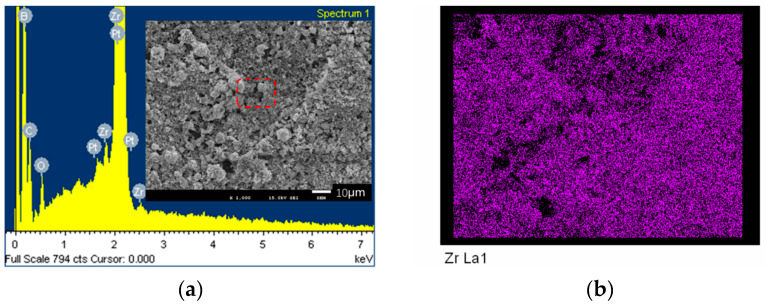

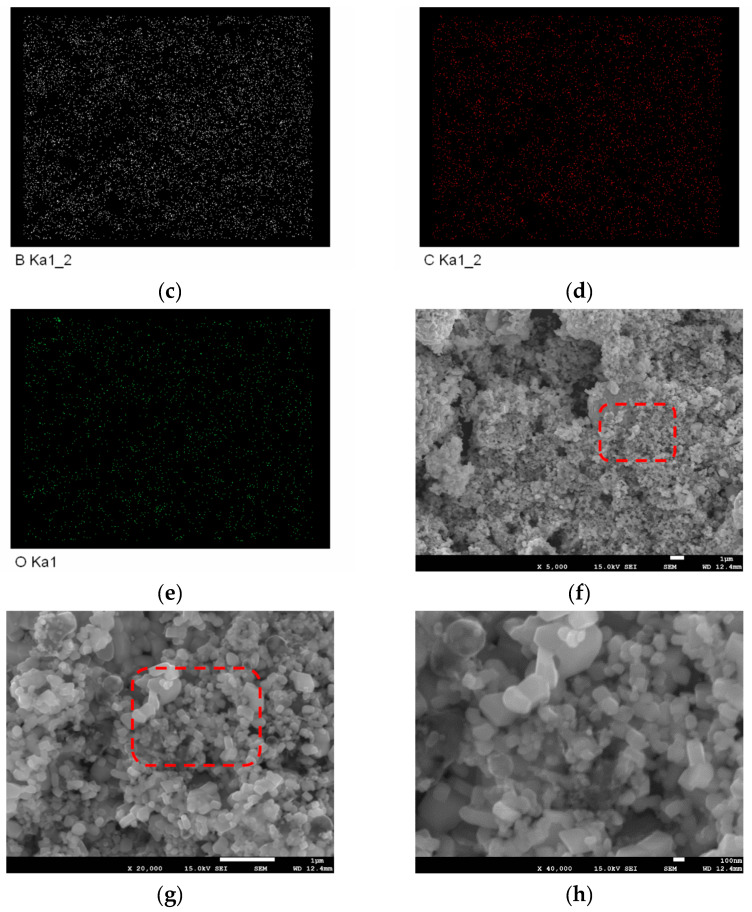
FESEM picture (**a**) and the corresponding EDS maps (**b**: Zr; **c**: B; **d**: C; **e**: O) of the RF thermal plasma synthesized ZrB_2_-ZrC composite powders, and the highly magnified FESEM picture (**f–h**).

Figure 7a shows a FESEM picture (insertion, magnified 1000 times) and a whole EDS spectrum of the RF thermal plasma synthesized ZrB_2_-ZrC composite powders when the mole ratio of C/B/Zr in the starting materials was set at 1:2:2. Figure 7b–e is the corresponding maps of the FESEM picture. It can be seen that the concentration of all main elements exhibits similar outlines with the FESEM picture, indicating that Zr, B, C, and O disperse uniformly in the sample. Figure 7f-–h shows FESEM pictures of the synthesized ZrB_2_-ZrC composite powders magnified 5000–40,000 times. The red circles are the magnified regions. The dispersed particles have clear edges and exhibit an average particle size ranging from 100 nm to 500 nm.

**Figure 8:**
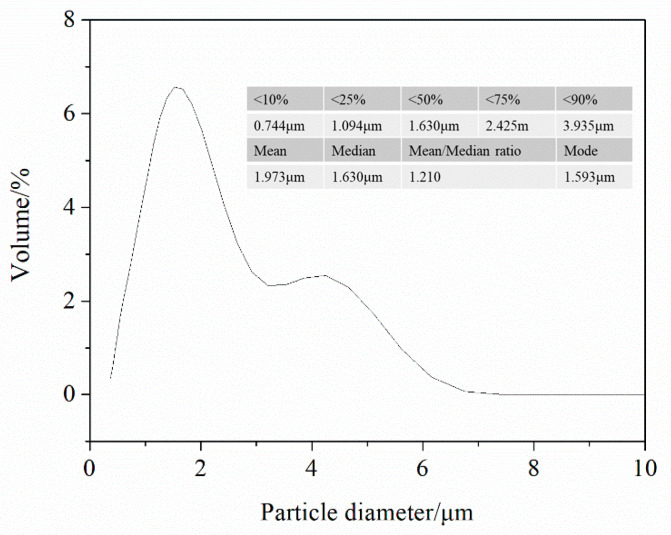
Particle size distribution of the RF thermal plasma synthesized ZrB_2_-ZrC composite powders.

The particle size distribution of the powders measured using an LS particle size analyzer (Beckman Coulter LS 13 320, Nyon, Switzerland) is shown in Figure 8. D10, D25, D50, D75, and D90 of the synthesized powder are 0.744 μm, 1.094 μm, 1.630 μm, 2.425 μm, and 3.935 μm, respectively. The mean diameter, the median diameter, and mode diameter are 1.973 μm, 1.630 μm, and 1.593 μm, respectively. The ratio of mean/median is 1.210. It can be seen that the particle size measured by the particle size analyzer is much higher than that detected under FESEM, which may be attributed to most of the particles aggregating together instead of being monodisperse.

The surface area of the synthesized ZrB_2_-ZrC composite powders calculated by BET was 32.15 m^2^/g, and the apparent density examined with the help of a Hall flowmeter was 0.57 g/cm^3^. Detailed structure characterization and further application of the RF thermal plasma synthesized ZrB_2_-ZrC composite powders are in progress.

## 4. Conclusions

An RF thermal plasma synthesis method was proposed for ZrB_2_-ZrC composite powders. Numerical simulation and thermodynamic analysis were conducted to predict the synthesis process, and experimental work was performed accordingly to demonstrate its feasibility. Several conclusions can be drawn from the results and discussion:

(1) ZrB_2_ was preferentially generated rather than ZrC, and free C instead of B would exist in the resulting products when C and B exceed the stoichiometric ratio.
(2) Solid raw materials could disperse well in the gaseous reactants, which leads to the uniform distribution of elements in ZrB_2_-ZrC composite powders.
(3) Free carbon particles can be removed during post-treatment, and ZrB_2_-ZrC composite powders with a particle size of about 100 nm could be obtained.
(4) The surface area of ZrB_2_-ZrC composite powders was 32.15 m^2^/g and the apparent density was 0.57 g/cm^3^.

## Figures and Tables

**Table 1:** Typical processing parameters for RF thermal plasma in our laboratory.

Numbers	Parameters	Values
1	Power supply	30 kW
2	Plasma gas (Ar)	2.0 m^3^/h
3	Sheath gas (Ar)	5.0 m^3^/h
4	Carrier gas (Ar/CH_4_)	0.2 m^3^/h
5	Feed rate (CH_4_)	0–0.6 L/min
6	Feed rate (H_2_)	0–0.3 L/min
7	Feed rate (solid)	4.0–16.0 g/min

**Table 2:** C and O contents of the products obtained with a different mole ratio of CH_4_/ZrCl_4._

CH_4_/ZrCl_4_	Carbon Content	Oxygen Content
0	0	6.06%
0.05	0.25%	3.91%
0.10	0.32%	3.45%

**Table 3:** Carbon and oxygen contents with different mole ratio of C/B/Zr.

C/B/Zr	Carbon Content	Oxygen Content
1:2:2	5.29%	3.5%
0.3:2:1	0.32%	3.45%
